# Antifungal Activity and Biochemical Profiling of Exudates from Germinating Maize Nostrano di Storo Local Variety

**DOI:** 10.3390/plants11182435

**Published:** 2022-09-19

**Authors:** Stefano Rosa, Stefano De Benedetti, Stefania Mazzini, Gigliola Borgonovo, Elisa Bona, Maria Cavaletto, Paola Antonia Corsetto, Martina Ghidoli, Salvatore Roberto Pilu, Alessio Scarafoni

**Affiliations:** 1 Department of Biosciences, University of Milan, 20133 Milan, Italy; 2 Department of Food, Environmental and Nutritional Sciences, University of Milan, 20133 Milan, Italy; 3 Dipartimento per lo Sviluppo Sostenibile e la Transizione Ecologica, University of Piemonte Orientale, 13100 Vercelli, Italy; 4 Department of Pharmacological and Biomolecular Sciences, Università degli Studi di Milano, 20122 Milano, Italy; 5 Department of Agricultural and Environmental Sciences—Production, Landscape, Agroenergy (DiSAA), Università degli Studi di Milano, 20133 Milano, Italy

**Keywords:** *Zea mays*, local varieties, germination, NMR, fatty acids, proteomics, endophytes, antifungal compound, plant protection, biodiversity

## Abstract

Plant pathogens are responsible for important damages to valuable crops causing important economic losses. Agrobiodiversity protection is crucial for the valorization of local varieties that could possess higher resistance to biotic and abiotic stress. At the beginning of germination, seeds are susceptible to pathogens attacks, thus they can release endogenous antimicrobial compounds of different natures in the spermosphere, to contrast proliferation of microorganisms. The work aimed at characterizing the maize of local variety Nostrano di Storo seed exudates secreted during the first phases of germination, to identify compounds active in the defense towards pathogens. Storo seed exudates were proven to inhibit *F. verticilloides* germination. In order to investigate the cause of the described effect, compositional profiling of the exudates was performed through NMR, lipidomic, and proteomic analyses. This study suggests an important role of microbial endophytic communities in the protection of the seed during the early phases of the germination process and their interplay with fatty acids released by the seeds, rather than a specific antifungal compound. The valorization of agronomically acceptable maize lines with pre-harvest enhanced resistances to pathogens contamination could lead, in the near future, to commercially available varieties potentially requiring more limited chemical protective treatments.

## 1. Introduction

Germination begins when a quiescent dry seed begins to absorb water (imbibition) and completes with the elongation of the embryonic axis. After imbibition, the seed resumes its metabolic activities, using the necessary structures and enzymes synthesized during seed development. The quick absorption of water leads to the rupture of the tegument and seed exudation, namely the dispersion of the internal substances outside the seed. This is due to the transition of the phospholipids of the tonoplast and plasmalemma from the gel state, reached during the desiccation of the seed, to the hydrated state.

The germinating seed and the surrounding soil (spermosphere) are excellent mediums for promoting growth and microbial interactions [1]. The exudates are a source of carbon and energy for microorganisms, facilitating microbial growth and the establishment of plant-microorganism interactions. Many released compounds can inhibit the growth of pathogens. The list includes organic molecules such as flavonoids, sterols and fatty acids [2], small peptides or whole proteins [3]. Fungal infections cause severe damage to crops during all production phases, from germination to post-harvest storage. Maize (*Zea mays)* is particularly vulnerable to mycotoxigenic fungi infections, such as *Fusarium verticillioides* [4]. The possible mycotoxin contamination can affect the quality and safety of food and feed, lowering the value of the grain and resulting in substantial economic losses [2].

Seeds at the early stages of germination are particularly susceptible to attack by pathogens. Chemical tanning is a surface protective treatment conducted directly on the seed with compounds or formulations with biocidal activity [5]. It has been used for many years to limit the growth of pathogens present in the soil or on the outer integument of the seed that could cause damage to the seed or to the developing seedling. However, tanning has significant environmental repercussions leading to a direct impact on human health through the contamination of raw materials of vegetable origin, and soil and water contamination. Moreover, in the long run, tanning impacts on soil fertility affecting the growth of beneficial microorganisms too [6,7].

To reduce the use of pesticides in agriculture, it is possible to resort to an eco-sustainable biocontrol action, using natural substances to counter the development of pathogens or using microorganisms that have an antagonistic activity towards pathogens, or choosing genotypes that can better adapt to the environmental conditions.

Endophytes, including fungi and bacteria, are microorganisms that colonize the internal tissues of plants without eliciting disease symptoms in crop plants. They can act as probiotics for their hosts contributing to host fitness and diversity; especially, bacteria can be isolated from surface-disinfected plant tissues or extracted from the host tissue [8]. The most comprehensive definition was proposed by Azevedo & Araujo [9], which defined an endophyte as a microorganism that may or may not be successfully cultured, that either internally colonize the host plant and do not cause apparent damage and/or visible external structures. So, the role of endophytic microbes in agricultural biotechnology ranges from mitigating environmental stressors to improving plant growth and health [10].

A variety of microorganisms have been isolated from surface-disinfected seeds, including endophytes and pathogens [11,12,13,14]. However, information regarding naturally occurring seed-specific endophytes and their plant interactions is limited [15,16].

One of the most reported seed endophytes is *Pantoea ananatis* that improve seed germination under salt stress in rice [17]. Moreover, *P. agglomerans* has been reported as an endophyte of different crop plants including pea [18], potato [19], sweet maize [20] and tomato [21]. Studies on the biocontrol of plant diseases using endophytic *P. agglomerans* have shown that it can control diseases such as anthracnose of cucumber [22], bacterial leaf spot of radish [23]. In addition, *P. agglomerans* was used as a surface-applied biocontrol agent for control of fungal diseases such as seedling blight of lentil caused by *Botrytis cinerea* [24], damping-off of safflower caused by *Pythium* spp. [25], white mold of bean caused by *Sclerotinia sclerotiorum* [26] and bean rust caused by *Uromyces appendiculatus* [27].

Biodiversity represents an infinite world of gene variability (and gene products) to draw on for the constitution of new varieties, with agronomically new characteristics, for the improvement of agriculture and the protection of the environment. Local varieties have enormous and still unexplored potential.

This work focuses on the seed exudates released by a local maize landrace, namely Nostrano di Storo, a municipality in the Trentino-Alto Adige region of Italy, and nine other maize landraces and varieties. The exudates have been tested for their capacity to inhibit *Fusarium verticilloides* conidial germination and characterized at the molecular level.

## 2. Results and Discussion

### 2.1. Nostrano di Storo Seed Exudates during Early Germination Inhibit F. verticilloides Conidial Germination

Seed exudation, namely the release of chemical compounds after seed imbibition, represents one of the first layers of defense against soil-borne diseases. Seeds own different weapons to counteract pathogens attacks, such as the presence of endophytic microorganisms [28], the release of antimicrobial molecules in the spermosphere [2], and the activation of latent enzymes [29]. To test the presence of antimicrobial factors in seed exudates from the selected maize local variety (Nostrano di Storo) during the first hours of germination, exudation was promoted by soaking seeds in water for two different time points; 32 and 72 h.

**Figure 1:**
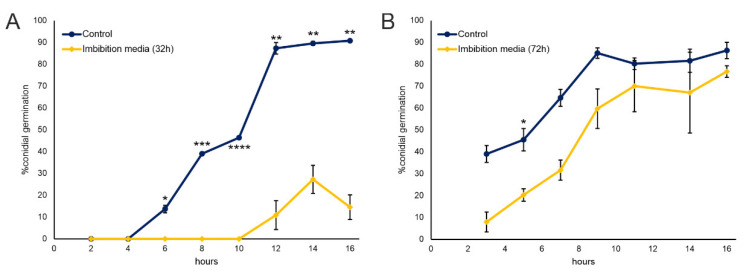
*F. verticilloides* conidial germination in presence of 32 h (**A**) and 72 h. (**B**) Nostrano di Storo seeds imbibition media. Results are reported as percent of germinated conidia with respect to total conidia ± standard deviation. Statistical differences were assessed with a Student’s T-test * *p* < 0.05, ** *p* < 0.01; *** *p* < 0.001; **** *p* < 0.0001.

Water incubated with and separated from seeds (from now on defined as “imbibition media”) was used to perform bioassays to test their capacity to inhibit the conidia germination of *F. verticilloides*, a widespread plant pathogen in Northern Italy and worldwide, capable to infect seeds at early stages of germination, causing seedling blight and other plant diseases [30]. Conidia suspensions were incubated with the imbibition media and % of conidial germination was determined every 2 h post-inoculation (hpi), for 16 h, and compared to untreated control. As shown in Figure 1A, 32 h imbibition media caused delayed germination of *F. verticilloides* conidia, sensibly decreasing their number with respect to the untreated control at 16 h. On the other hand, the conidia treatment with the imbibition water recovered after 72 h of incubation with maize, which showed a germination curve more similar to the control (Figure 1B).

These observations indicate the presence of antimicrobial compound(s) able to delay the germination of *F. verticilloides*, transiently released by the germinating maize seeds.

To test if the observed antimicrobial activity is genotype and/or environmentally dependent, other local varieties cultivated in the same experimental fields in Landriano (45°18′41″ N, 9°15′37″ E), were taken into consideration as reported in Table 1.

**Figure 2:**
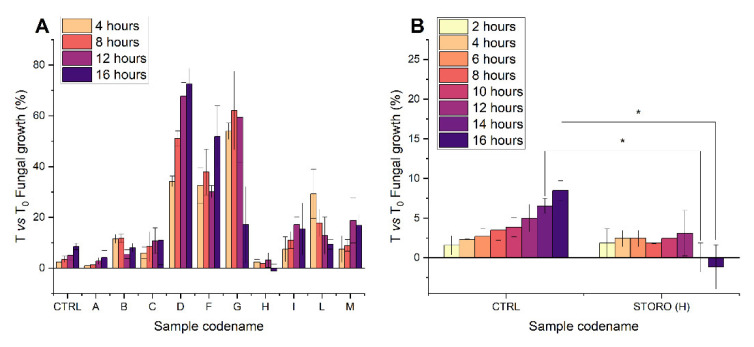
*F. verticilloides* fungal growth in presence of imbibition media of selected maize local varieties, grown in the same fields (**A**). Data are reported as % absorbance increase at each timepoint with respect to initial timepoint ± standard deviation. (**B**) Reports the investigation at closer timepoints of fungal growth in presence of sample H (Nostrano di Storo maize local variety) in comparison with a control sample where the same amount of fungal conidial were incubated without any maize imbibition medium. Statistical significance was assessed with ANOVA test * *p* < 0.01.

An equal number of seeds (200) were soaked in water for 32 h and imbibition media were recovered to perform the antimicrobial assays. Mycelium development was monitored by measuring absorbance increase in a multi-well plate at 492 nm either in presence of 32 h of seed exudates, or with an untreated sample. The growth rate was determined at each timepoint as the % absorbance increase with respect to the initial absorbance (T_0_) [31]. Growth rate varies in dependence of the ecotype exudate tested (Figure 2A) but only Nostrano di Storo exudate is able to inhibit conidia germination and mycelium development. It is worth noting that its inhibition remains significant during the whole duration of the experiment (Figure 2B).

These results indicate that Nostrano di Storo exudate possesses the strongest antifungal activity, thus it seems that this bioactivity could be more related to a peculiarity of this local variety than to environmental influences. In light of these results, we decided to more deeply investigate the composition of Nostrano di Storo exudate in order to identify the potential antifungal compound(s).

### 2.2. NMR Nostrano di Storo Exudation Profiles Over-Time

**Figure 3:**
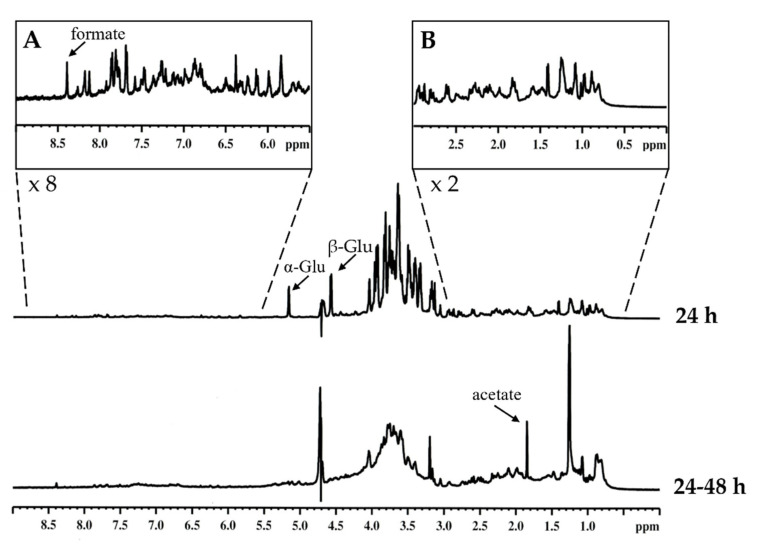
^1^H NMR spectra of maize Nostrano di Storo exudate dissolved in D_2_O at 25 °C. In the upper panel the spectra related to the first 24 h after imbibition are reported; in the lower panel, the second interval of 24 h after replacing medium (24–48 h) is reported. In the insets are presented the magnifications of the high field regions attributable to aromatic/olefinic compounds (**A**) and low field regions attributable to aliphatic compounds (**B**) of the exudate after 24 h of imbibition with the relative magnification power.

We used ^1^H NMR analysis to indicatively identify the main components and the differences in the relative proportion of various classes of organic compounds from seed’s exudate of Nostrano di Storo maize local variety in the first 24 h and between 24 and 48 h after imbibition (Figure 3). The region between 3.0 and 4.1 ppm, attributable to signals carbohydrates protons [32], was characterized by an overlapping of the signals. Similarly, it was possible to distinguish the anomeric protons of the α and β forms of glucose (5.16 ppm, J = 3.60 Hz and 4.58 ppm, J = 7.92 Hz, respectively). For all considered samples, the ratio of the integrals of α and β signals remains constant (36% for α and 64% for β), typical values of equilibrium between the anomeric forms in aqueous solution. The high-field region (0–3.0 ppm) is characterized by broad and poorly resolved signals, typically attributable to aliphatic systems of amino acid and/or fatty acids chains [32,33]. The low-field region (5.5–9.0 ppm) showed broad signals, indicating a low content of olefinic and aromatic protons, possibly attributable to polyphenolic compounds. All samples showed the presence of an intense singlet at 8.4 ppm likely attributable to formate [32]. It is intriguing to note that a signal at 1.9 ppm is particularly evident between 24 and 48 h. (Figure 3). This peak could be attributable to acetate, which, together with formate, are products of fermentation [34]. One hypothesis could be that this fermentation could occur due to the presence of seedborne endophytic bacteria.

The imbibition medium composition of Nostrano di Storo seeds was compared to a commercial hybrid exudate obtained in the same conditions, namely hybrid B73/Mo17, which is one of the most common past commercial hybrids currently used in various agronomic and genetic research projects [35].

The relative proportion of different components in the two samples was calculated by integration, after subtracting the integral area of the solvent peak (Table 2). The higher proportion was represented by carbohydrate compounds, followed by aliphatic systems of amino acid and/or fatty acids chains. The lower proportion was represented by olefinic and aromatic species. In the sample related to the second 24 h of exudation, the spectra displayed a line broadening, suggesting different equilibria in solution involving the formation of different species in the NMR time scale.

By the analysis of the data reported in Table 2, a marked increase in aliphatic compounds in Nostrano di Storo exudate (+84%) emerged if compared to the commercial reference (+13%), while the decrease in carbohydrates from 24 to 48 h of the exudation is very similar among samples. The most relevant increase in metabolites released in Nostrano di Storo exudate would be probably attributable to the class of aliphatic compounds. The data, as a whole, albeit with the limits of the methodological approach adopted, strongly suggested the release of fatty acids and/or amino acids in the exudates. We thus decided to focus our attention on these two classes of compounds performing lipidomic and proteomic analyses.

### 2.3. Free Fatty Acid Analysis of Maize Seeds and Antifungal Active Exudate

To further investigate the molecular composition of 32 h imbibition medium, which showed the highest antifungal activity, we evaluated the fatty acid profile through Methyl esters fatty acids (FAME) analysis, by comparing the exudate with the Nostrano di Storo seed lipid content. Fatty acids have a crucial role in the interactions that take place between plants and both beneficial and/or pathogenic microbes. Increased levels of palmitoleic acid (C16:1) indeed, were described to promote resistance to *Verticillium dahlia* in eggplants [36]; the ratio between oleic (C18:1) and linoleic (C18:2) acid is correlated with soybean seed colonization by the pathogen *Cercospora kikuchii* [37], while free linoleic acid has been shown to influence *Aspergillus* spp. mycotoxin production [38]. Finally, the positive interaction that takes place with arbuscular-mycorrhizal fungi depends on plant derived free palmitic acid (C16:0) [39,40].

**Figure 4:**
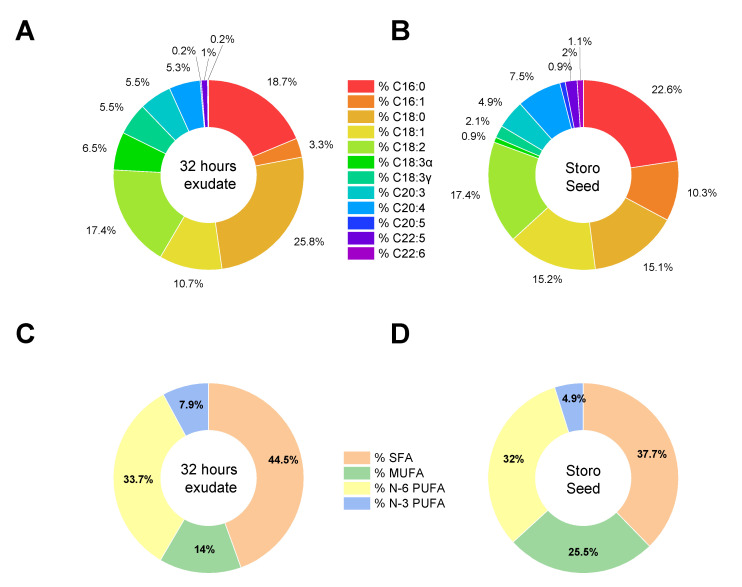
Methyl esters fatty acids (FAME) on 32 h Nostrano di Storo seed imbibition medium (**A**,**C**) and on ungerminated Nostrano di Storo seeds (**B**,**D**). Panel A and B report the relative proportion of the identified FAME, while panels C and D group FAME according to the saturation of the aliphatic chains as Saturated Fatty Acids (SFA); Monounsaturated Fatty Acids (MUFA); ω-6 Polyunsaturated Fatty Acids (N-6 PUFA); ω-3 Poly Unsaturated Fatty Acids (N-3 PUFA).

The major free fatty acids detected in analyzed seeds are palmitic C16:0 (22.6%), linoleic acid C18:2 (17.4%), oleic C18:1 (15.2%); stearic C18:0 (15.1%), and palmitoleic C16:1 (10.3%) (Figure 4). Saturated fatty acids (SFA) account for about 37.7%, while monounsaturated fatty acids (MUFA) represent the 25.5% and polyunsaturated fatty acids the 36.8% of the total fraction, with an overall ratio SFA/UFA of 0.6. When analyzing the FAME profile of exudate, the most striking finding was the high presence of stearic acid C18:0 (25.8%), with respect to the other FA, considering that it is not the most represented FA in the seed. This occurrence, together with a reduced amount of MUFA (14.0%), in particular palmitoleic acid C16:1 (3.3%), leads to an increase in the proportion between SFA and UFA in the 32 h imbibition medium to 0.8. The high amount of stearic acid suggests an active release of this particular saturated fatty acid, that could underline a potential role in seed development and/or defense. Moreover, the first lipase activity in maize is documented after 2–3 days of germination and its activity is preferentially directed towards oleic and linolenic acid containing triacylglycerols [41]. The stearic acid released in the exudates should thus originate from the seed’s stored supply.

Fatty acids and their derived compounds are key mediators of interkingdom communication [42]. In addition, one of the key steps in regulating UFA levels in cells is the conversion of stearic acid (C18:0) to oleic acid (C18:1) [43]. The plant-pathogen interaction may be mediated by FAs themselves as well as by PUFAs derivatives, known as oxylipins [35]. Linoleic acid (C18:2), PUFA derived from stearic acid, has been described to be involved in wheat resistance to infection from *F. graminearum*, possibly by reinforcing cuticle, strengthening the barrier against pathogen entry [44].

PUFAs are converted into fatty acid hydroperoxides (oxylipins) through lipoxygenase activity and are crucial in plant-pathogen interaction. Indeed, plant oxylipins interfere with fungal development mediated by the same class of molecules, probably by mimicry. In a study investigating lipoxygenase activity in maize—pathogen interaction, Gao et al., demonstrated that *F. verticillioides*—infected kernels exhibited increased fatty acids, 7 days post infection, specifically, C16:0, C18:0, C18:1 (including both 9Z and 11Z isomers), C18:2, and C18:3 [45].

Recently, it was reported that palmitic acid inhibited mycelia growth of *F. oxysporum* by changing the rhyzosphere microbial composition, finally reducing the severity of Fusarium wilt in watermelon [46]. Furthermore, UFA, SFA and their derivatives, such as oxylipins, have been reported as antifungal agents, not only for their potential to inhibit fungal growth, but also for their ability to impair mycotoxin production. They could be used as promising natural and environmentally friendly compounds to control plant disease, while being less likely to promote fungal resistance [47]. For all these reasons identifying *Zea mays* ecotypes, naturally resistant to fungal infections through these mechanisms, could be of paramount importance for crop valorization.

In our analyses, levels of C18:0 and C18:3 in 32 h exudation medium, stand out from those of others fatty acids, suggesting that Storo maize could naturally release these fatty acids that could represent protective factors against *F. verticilloides* infection.

### 2.4. Characterization of the Protein Fraction

After focusing on fatty acids, we proceeded to evaluate the proteomic profile of the imbibition medium since proteins are well-known antifungal molecules [48]. Previous similar studies, aimed at characterizing lupin protein exudate profile, led to the identification of polypeptides involved in seed defense. These were demonstrated to be actively produced and secreted, after a first passive extrusion phase, during the early hours of germination. In particular, lupin exudate was able to inhibit *A. niger* and *P. expansum* growth [3].

The protein composition of the Nostrano di Storo exudates released after 32 h of germination was thus characterized. Protein fraction was separated using preparative SDS-PAGE. Ten slices spanning the whole molecular weight separation range were excised from the gel slab and subjected to mass spectrometry analyses (Table S1).

At the investigated timepoint, surprisingly no proteins originating from the germinating seeds were identified. The analysis revealed instead the presence of proteins belonging to several bacterial species, most of them were attributable to seeds’ endophytic communities.

Among the 12 peptides identified with mass spectrometry indeed, six belonged to proteins that could be annotated as originating from the genus *Pantoea*, in particular *Pantoea ananatis*. In addition, five peptides belonged to different *Enterobacteriaceae*, common environmental bacterial species.

Genus *Pantoea* comprises common endophytic bacteria of maize seeds, often associated with specific cultivar; *Pantoea ananatis* is one of the most representatives [49,50]. The latter indeed, has been demonstrated to exert antibacterial and antifungal activity both in vivo and in vitro, thus having the potential to protect seeds from pathogens. Its beneficial role as an endophyte has been described by several authors [51,52] and its antifungal activity is common to other endophytes able to enhance plant resistance against *F. graminearum* as well as plant growth [53]. Finally, *Pantoea agglomerans* is able to produce d-alanylgriseoluteic acid, a potent antimicrobial phenazine compound [54].

Interestingly, among the proteins identified, three sequences belonged to different translation elongation factor Tu (EF-Tu), all sharing a high degree of similarity, as assessed through alignment analyses, performed with Tcoffee (Supplementary Figure S1). EF-Tu exerts its main activity in the protein synthesis, however other secondary still underexplored functions have been described. EF-Tu indeed, can be exposed on the bacterial surface, thus relating its activity to virulence traits in pathogenic prokaryotes [55]. Other EF-Tu functions are associated with adherence to host molecules and to cytoskeleton components [56]. However, more interestingly, the first twelve amino acids of EF-Tu are able to suppress the apoptotic response in plant cells triggered by Fumonisin B1. The same authors obtained the same results by incubating cells with a peptide originating from bacterial flagellin protein, showing that pattern-triggered immunity suppresses programmed cell death elicited by Fumonisin B1 [57]. In addition, two proteins identified in this work belong to flagellin family and originate from *Pantoea* spp.

## 3. Materials and Methods

### 3.1. Seed Samples and Imbibition Media Collection Procedures

Maize seeds were obtained from Prof. Pilu and to each variety was attributed a letter codename as reported in Table 1. All the local varieties were cultivated in the same experimental fields in Landriano (45°18′41″ N, 9°15′37″ E) in the same year. Maize kernels were surface sterilized with sodium hypochlorite 0.5% and extensively washed with sterile water. Seeds were incubated under mild shaking in distilled water (1 mL/seed) at 25 °C. The media, when removed for timepoint analyses at 24 or 32 h after imbibition, were replaced with sterile distilled water. For each *Zea mays* variety, the experimental point was collected in two different tubes, the contents of which were pooled at the end of the incubation time. At the end of the incubation time, the imbibition media were immediately centrifuged at 10.000 rpm for 30 min to remove any insoluble material and frozen at −80 °C until analyzed.

### 3.2. Antifungal Activity Assay

In order to quantify the Nostrano di Storo imbibition medium inhibitory activity on the germination of *F. verticilloides* conidia, direct count under the optical microscope was used as evaluation method as reported in Passera et al. [58]. Briefly, freshly harvested conidia at a concentration of 10^4^ conidia/mL were tested in triplicate for germination either alone in control medium Potato Dextrose Broth (Sigma-Aldrich; St. Louis, MO, USA), or in presence of 32 h, and 72 h imbibition media. The test tubes were incubated at 24 °C during the day and 10 °C at night, readings were taken every 2 h for 16 h. Germination was evaluated by direct observation under an optical microscope (20×; Easylab CX40, Olympus, Shinjuku, Tokyo, Japan) using a Kova counting grid, considering each spore to have germinated if the length of germination tube was twice as long as the conidium diameter [59]. For each observation, 100 spores were visually analyzed and determined to be either germinated or non-germinated. Conidial germination rate (GR) was calculated as (G/C) × 100, where G is the number of germinated conidia detected, and C is the total number of conidia counted.

The screening of seed imbibition media biological activity from the others’ considered local varieties, was assessed in 96-well microplates (Sero-wel, Bibby Sterilin Ltd., Stone, UK) in three replicas, according to the quantitative method proposed by Raposo et al. [31]. Isolates of *Fusarium verticilloides* (Sacc.) stored in potato dextrose agar slants kept at 4° C, were grown on the same medium in Petri dishes for 7 days at 24 °C. Conidial suspensions were prepared by transferring a loop of mass spores differentiated on the mycelium surface in a 1.5 mL tube containing sterile potato dextrose broth at double concentration (PDB 2×) and adjusted at the final count of 2 × 10^4^ conidia/mL. Wells were filled with 50 µL of conidial suspension and 50 µL of seed exudate. Each exudate was tested in triplicate, as a control, sterile distilled H_2_O was added in place of the samples. The absorbance of each well was measured at 492 nm wavelength using a SunriseTM Absorbance Reader (Tecan Group Ltd., Männedorf, Switzerland) immediately before (T_0_) and each two hours of incubation (T_x_), at 24 °C in the dark up to 16 h. The growth of *F. verticilloides* was quantified in treated and untreated wells by calculating the percent absorbance increase at each timepoint vs. T_0_. The fungal growth was calculated according to the formula (1):

(1) $$\frac{\left({AT}_{x}-{AT}_{0}\right)}{\left({AT}_{0}\right)}*100$$

where AT_0_ is the initial absorbance and AT_x_ is the absorbance of the treated sample at each timepoint.

### 3.3. NMR Analyses

NMR spectra were recorded on a Bruker AV600 (Billerica, MA, USA) spectrometer operating at a frequency of 600.10 MHz for ^1^H equipped with a z-gradient 5 mm reverse probe. The ^1^H NMR spectra were recorded at 25 °C. Chemical shifts (δ) were measured in ppm and referenced to external 2,2-dimethyl-2-silapentane-5-sulfonate sodium salt set at 0.00 ppm. For NMR experiments, the samples were dissolved in D_2_O. Solvent suppression was achieved by presaturation with the carrier placed on the water resonance.

The NMR analysis on seed samples germinated for 24 h, and on samples in which the growth medium was replaced after 24 h, in order to analyze the exudation between 24 and 48 h. The same kind of test has also been conducted considering samples of hybrid maize B73/Mo17, prepared with the same methodology.

The ^1^H NMR spectra were divided into 3 regions: 0–3.0 ppm (H in straight chain, branched, and cyclic alkanes, CH_3_COOR); 3.0–5.5 ppm (H in N-alkyl-CH, O-alkyl-H, sugar); 5.5–9.0 ppm (aromatic H or olefinic H). The relative proportion of different components in all samples was calculated by integration, after subtracting the integral area of the solvent peak. For each sample, three spectra were recorded. The accordance of area determination of integrated regions was 5–8%. NMR spectra were elaborated by using TOPSPIN 1.3 software (Bruker BioSpin GmbH, Rheinstetten, Germany).

### 3.4. Lipid Extraction and Analyses

Sample lipids were extracted with three different chloroform/methanol mixtures 1:1, 1:2, 2:1 (*v/v*). Each solvent contained 50 μM 2,6-bis(1,1-dimethylethyl)-4-methylphenol (BHT) to protect lipids from oxidation. The organic phase was dried and suspended in chloroform/methanol (2:1) for the analysis of total fatty acid (FA) composition. Total FAs were determined as methyl esters (FAMEs) by gas chromatography. The methyl esters were obtained by reaction with 3.33% (*w/v*) sodium methoxide in methanol and injected into an Agilent Technologies (6850 series II) gas chromatograph (Santa Clara, CA, USA) equipped with a flame ionization detector (GC-FID) and a capillary column (AT Silar) (length 30 m, film thickness 0.25 μm). The carrier gas was helium, the injector temperature was 250 °C, the detector temperature was 275 °C, the oven temperature was set at 50 °C for 20 min and then increased to 200 °C at 10 °C min^−1^ for 20 min. A standard mixture containing all FAMEs was injected for calibration, and TG C17:0 was added and used as an internal standard.

### 3.5. Mass Spectrometry

SDS-polyacrylamide gel electrophoresis (SDS-PAGE) was conducted according to Laemmli [60]. Protein samples were denatured, in the presence of β-mercaptoethanol, by 10 min heating at 100 °C in the SDS-PAGE sample buffer and loaded into SDS gels. Run was carried out at 16 mA constant for each gel. After electrophoresis, the protein bands were visualized using Coomassie Blue G-250 staining (BioRad, Milan, Italy). The Mr markers were: β phosphorilase (92 kDa), BSA (66 kDa), egg’s albumin (45 kDa), carbonic anidrase (30 kDa), trypsin inhibitor (20 kDa) and lisozyme (14 kDa) (Bio-Rad, Hercules, CA, USA).

A gel slab was cut into ten slices to and destained overnight with a solution of 25 mM ammonium bicarbonate and 50% acetonitrile. The proteins were digested with trypsin (Roche, Segrate, Milano, Italy) in-gel digested as described by Hellmann et al. [61]. All nano-HPLC-MS/MS experiments were performed on a Q-TOF mass spectrometer Q-Star XL (AB Sciex, Concord, Ontario, Canada) controlled by the Analyst QS 1.1 software version 5.0.2195 (AB Sciex, Washington, DC, USA) connected to an Ultimate 3000 nano-HPLC system. The precipitated pellets were resuspended in 10 μL of solvent A (95% *v/v* water, 5% *v/v* acetonitrile, 0.1% *v/v* formic acid). Five microliters of each sample were loaded onto the precolumn, 300 μm i.d. × 5 mm, C18 PepMap, 5 μm beads, 100 Å, (LC-Packings) and washed for 5 min using a flow rate of 40 μL min^−1^ solvent A. The peptides were subsequently eluted at 300 nl min^−1^ from the precolumn over an analytical column, 15 cm × 75 μm, C18 PepMap100, 3 μm beads, 100 Å (LCPackings) using a 35 min gradient from 5 to 60% solvent B (5% *v/v* water, 95% *v/v* acetonitrile, 0.1% *v/v* formic acid) delivered at 300 μL min^−1^. The analytical column was connected with a 15 μm inner diameter Silica Tip (Pico Tip) nanospray emitter (New Objective, Woburn, MA, USA). The spray voltage (set between 1800 and 2000 V) was applied to the emitter through a stainless-steel union and tuned to get the best signal intensity using a standard BSA tryptic digest before every sample’s batch submission. The QStar-XL was operated in information-dependent acquisition (IDA) mode. Mass spectra were acquired from 400 to 1800 m/z. The two most intense ions with charge states between 1 and 4 in each survey scan were selected for the MS/MS experiment. MS/MS data were acquired from 60 to 1800 m/z. Each acquisition cycle was comprised of a 1 s MS and a 3 s MS/MS. The MS to MS/MS switch threshold was set to 15 counts per second (c.p.s.). All precursor ions subjected to MS/MS in the previous cycle were automatically excluded for 60 s using a 3 amu.

### 3.6. Statistical Analyses and Software

Statistically significant differences were assessed by using Student’s T-test and ANOVA, where appropriate, by using the software Origin (Pro), Version2021b (OriginLab Corporation, Northampton, MA, USA). The same software and CorelDraw2020 were used to prepare graphical representation of data. Multiple Sample Alignment was performed by using T-Coffee Web Service through Jalview 2.11.1.0, [62,63] available at https://www.tcoffee.org/ (accessed on 8 April 2022) and http://www.jalview.org/jalview-js/ (accessed on 8 April 2022), respectively.

## 4. Conclusions

The work aimed at characterizing the seed exudates secreted during germination in order to identify compounds active in the defense towards pathogens.

With this work we can conclude that the maize Nostrano di Storo presents valuable characteristics that should valorize this local variety, such as resistance towards infection from *F. verticilloides*. Our results strongly indicate that this characteristic is a peculiarity of this variety and it is not attributable to growth conditions, since other varieties that have been grown exposed to the same environmental stressors do not present this resistance. The reasons behind this antifungal activity must still be addressed, however we can hypothesize that molecules produced by the seeds such as fatty acids could play a defensive role. Another hint points towards the role of endophytic bacteria, in particular belonging to the *Pantoea* spp. that have been shown to colonize the seeds of Nostrano di Storo maize, used in this study. It is more likely that an interplay between endophytic bacteria and plant produced molecules, such as fatty acids, is the key to the resistance to pathogens attack. These molecules could not be the only actors in this interaction, peptides indeed could possess interesting antifungal properties [64], however with this experimental setup we did not analyze this aspect that will be addressed in future work.

Finally, the valorization of agronomically acceptable maize lines with pre-harvest enhanced resistances to aflatoxin and fumonisins contamination could lead, in the near future, to commercially available varieties potentially requiring more limited chemical protective treatments. To make this possible, it is necessary to identify the compounds at the basis of these properties, and the genetic components that determine them. Crossbreeding programs will allow to identification of new varieties expressing at high levels the molecules able to defend the plant.

## Figures and Tables

**Table 1:** List and relative codename of the analyzed maize local varieties.

SAMPLE	NAME
A	B73/Mo17
B	R2869 RP1 x BP1
C	R2830 Biancoperla
D	R2818 Cinquantone
F	R3303 Pignoletto
G	R2826 Bianco Vitreo
H	R2833 Nostrano di Storo
I	R2828 Nostrano dell’isola
L	R3304 Ottofile tortonese
M	R2822 Marano

**Table 2:** Relative proportion (%) of the components obtained by ^1^H NMR analysis and relative fold change in the analyzed time period.

Samples	Aliphatic H (0–3.0ppm)	O-Alkyl H (3.0–5.5 ppm)	Aromatic/Olefinic H (5.5–9.0 ppm)
Nostrano di Storo			
24 h (%)	19.00	80.28	0.72
24/48 h (%)	35.01	64.51	0.47
Fold change	1.84	0.80	0.65
B73/Mo17			
24 h (%)	35.83	59.03	4.13
24/48 h (%)	40.45	48.41	11.14
Fold change	1.13	0.82	2.70

## Data Availability

Not applicable.

## Supplementary Materials

The following supporting information can be downloaded at: https://www.mdpi.com/article/10.3390/plants11182435/s1, Figure S1. Multiple Sequence Alignment of the three Ef-TU proteins identified in the 32 h imbibition medium through MS analyses. Table S1: List of the identified proteins by Mass Spectrometry.
