# Supplementary material for: Antifungal Activity and Biochemical Profiling of Exudates from Germinating Maize Nostrano di Storo Local Variety
Source: Plants (Basel). 2022 Sep 19;11(18):2435. doi: 10.3390/plants11182435 (PMC9505497; doi:10.3390/plants11182435)
Supplement: Supplementary file 1 [file plants-11-02435-s001.zip › plants-1878423-supplementary.pdf]

Table S1: List of the identified proteins by Mass Spectrometry. In red are reported those attribution that failed statistical analysis significance test.

Lane	Accession	Name	Organism	Mass (Da)	Score	Num. Of peptides
1	WP_010616442	elongation factor G	*Plautia stali symbiont*	77503	326	7
	WP_013357316	30S ribosomal protein S1	*Pantoea*	61162	155	3
	WP_010618846.1	glycogen-debranching protein	*Plautia stali symbiont*	78320	125	2
2	WP_007889058	phosphoenolpyruvate-protein phosphotransferase	*Pantoea sp. GM01*	63010	71	1
3	WP_012368508.1	elongation factor Tu	*Proteus mirabilis*	43169	120	2
4	WP_158783929	class II fructose-bisphosphate aldolase	*Pantoea sp. BAV 3049*	39160	40	1
5	WP_239313728.1	FliC/FljB family flagellin	*unclassified Pantoea*	37919	85	1
	NJQ18861.1	cysteine synthase A	*Pantoea sp. LS15*	34482	63	1
6	ELU41973.1	pinin/SDK/memA conserved region domain-containing protein	*Rhizoctonia solani AG-1 IA*	32445	41	1
7	WP_013493728	23S rRNA (guanosine(2251)-2'-O)-methyltransferase RlmB	*Intrasporangium calvum*	33582	50	1
8	ND					
9	AFS33537	elongation factor Tu, partial (chloroplast)	*uncultured Ulvophyceae*	25188	123	2
	ABV45527.1	translation elongation factor Tu, partial	*Pantoea ananatis*	30116	66	1
10	WP_013026158.1	flagellin	*Pantoea ananatis*	32234	123	2
	WP_003834654	porin OmpC	*Citrobacter freundii complex*	40536	150	2
	WP_010618757	glyceraldehyde-3-phosphate dehydrogenase	*Plautia stali symbiont*	35511	72	2

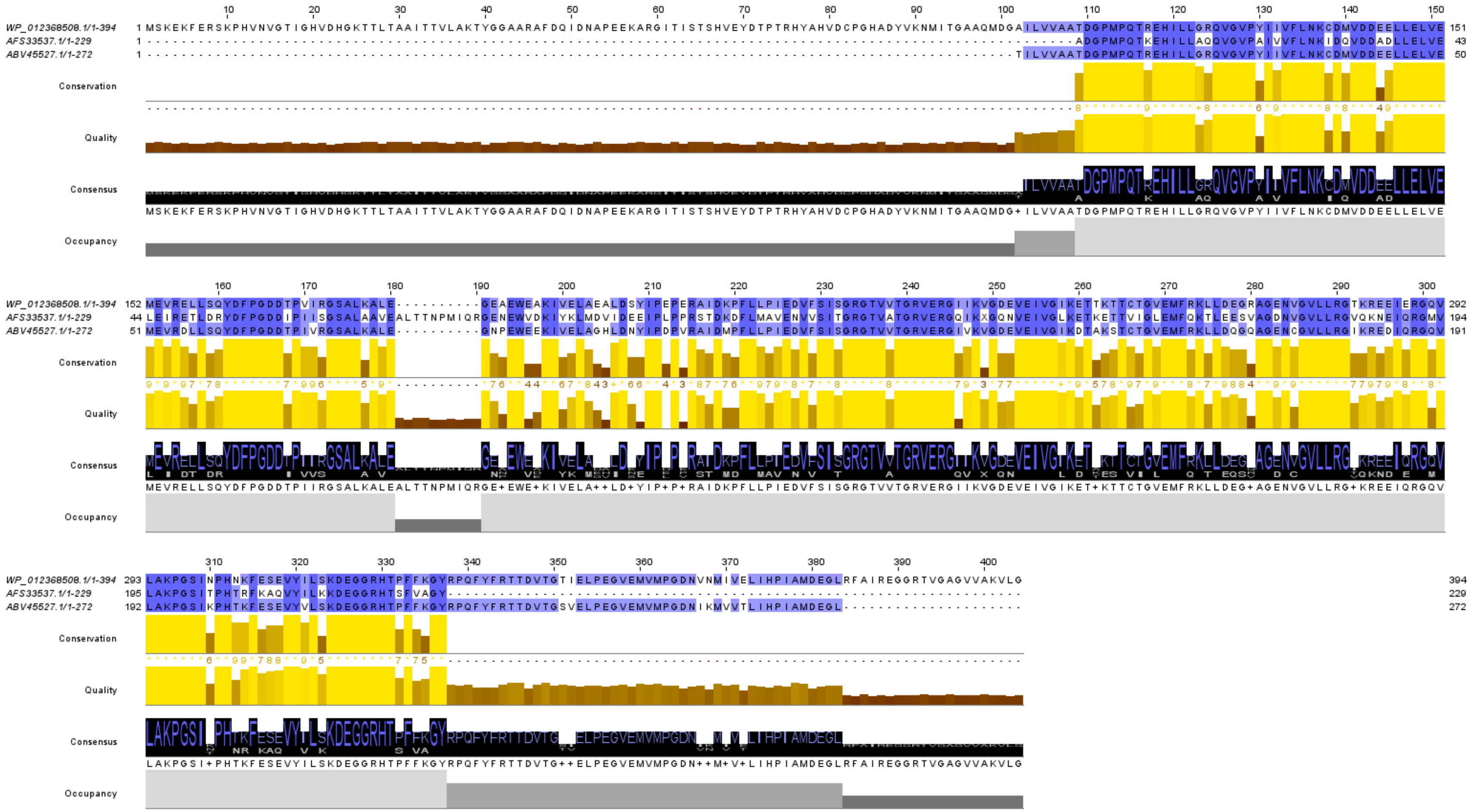

Figure S1. Multiple Sequence Alignment of the three Ef-TU proteins identified in the 32 hours imbibition medium through MS analyses. WP_012368508.1 elongation factor Tu [*Proteus mirabilis*]; AFS33537.1 elongation factor Tu, partial (chloroplast) [uncultured Ulvophyceae]; ABV45527.1 translation elongation factor Tu, partial [*Pantoea ananatis*]. Residue conservation is reported as histogram bar while sequence coloring represents percentage identity; conservation and consensus logos are shown.
